# Synthesis of a novel aminobenzene-containing hemicucurbituril and its fluorescence spectral properties with ions

**DOI:** 10.3762/bjoc.17.195

**Published:** 2021-12-06

**Authors:** Qingkai Zeng, Qiumeng Long, Jihong Lu, Li Wang, Yuting You, Xiaoting Yuan, Qianjun Zhang, Qingmei Ge, Hang Cong, Mao Liu

**Affiliations:** 1 Department of Chemistry, College of Chemistry and Chemical Engineering, Guizhou University, Guiyang, Guizhou Province 550025, PR China

**Keywords:** amidobenzene-containing macrocycle, hemicucurbituril, host–guest interaction, macrocycle, modification

## Abstract

A novel hemicucurbituril-based macrocycle, alternately consisting of amidobenzene and 2-imidazolidione moieties was designed and synthesized. Based on the fragment coupling strategy, nitrobenzene-containing hemicucurbituril was firstly prepared facilely under alkaline environment, and reduction of the nitro groups produced the desired amidobenzene-containing hemicucurbituril. As an original fluorescent chemosensor, it exhibited strong interactions with Fe^3+^ over other metal cations. The experimental evidence of fluorescence spectra suggested that a 1:1 complex was formed between this macrocycle and Fe^3+^ with an association constant up to (2.1 ± 0.3) × 10^4^ M^−1^. Meanwhile, this macrocycle showed no obvious or only slight enhancement of the fluorescence intensity with selected anions.

## Introduction

Macrocycles with converging binding sites and functional groups hold a key position in supramolecular chemistry, which has been repeatedly confirmed by classic macrocyclic molecules, such as crown ethers, cyclodextrins, calixarenes, cucurbiturils and their homologues [1]. For the past decades, numerous intriguing macrocycles have come into our sight, cycloparaphenylenes [2–8], pillararenes [9–12], tiaraarenes [13–14], coronaarenes [15–17], heteracalixaromatics [18–19], and hemicucurbiturils [20] for instance, and these modified macrocycles have been applied into practical domains, such as chemosensors [21], drug delivery [22], and nano materials preparation [23].

**Scheme 1:**
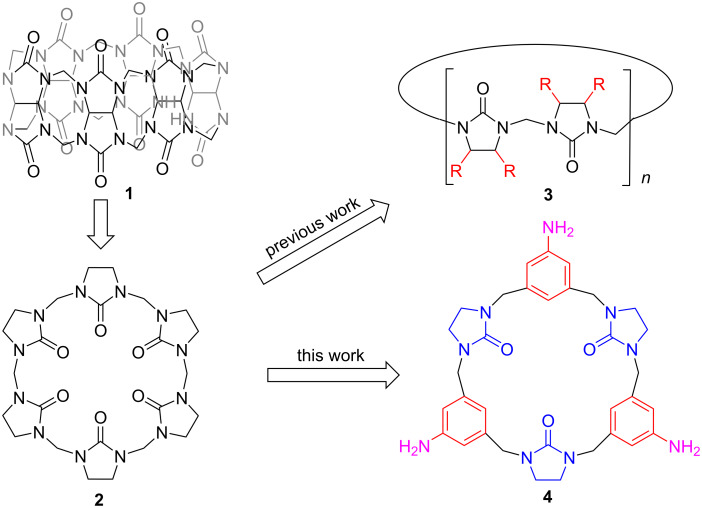
The evolution of hemicucurbituril analogs.

Although cucurbiturils with their rigid hydrophobic cavities have found broad application in host–guest chemistry, they suffer from insolubility, difficulty in derivatization, and lack of chromophores. Hemicucurbit[*n*]urils (HQ[*n*]) and relevant derivatives [24] represent as a sub-group of the cucurbituril family [25], possessing much more flexible structures than Q[*n*]s, and are generally characterized by an electroneutral cavity and a negative electro-potential portal. Comparing the coordination properties of HQ[*n*]s with that of Q[*n*]s, Buschmann [26] observed that HQ[6] **2** (Scheme 1) formed complexes only with Ni^2+^, Co^2+^, and UO_2_^2+^ with extremely low affinity, which may be caused by the poor solubility of HQ[6] **2** in aqueous solution and its universal “alternate” conformation. Most modified hemicucurbiturils **3** (Scheme 1) have been template synthesized [27] in succession by modified imidazolidiones, including bambusurils [28–30], cyclohexylhemicucurbiturils [31], and semithiobambusurils [32]. On the other hand, in previous works, authors discussed the ability of the derivatives for accommodating ions at submicromolar concentrations always by means of NMR spectroscopy [33]. Nevertheless, sensing by UV–vis or fluorescence spectra was hard to realize, for there is no chromophore in the frameworks. While hemicucurbit[*n*]urils with improvement in solubility, they still remain poor in derivatization. These adverse properties have impeded the development of hemicucurbit[*n*]urils to some extent.

Introducing aromatic fragments into macrocycles which have no chromophore is a viable strategy to expand the scope of their application. Ganin et al. [34] reported the synthesis and formation of several heterocalixarenes, which all comprised benzimidazol-2-one and 1,3-phenylene units in an alternate cyclic arrangement. In 2008, Kwit et al. [35] synthesized urea and thiourea derivatives of chiral triangular polyimine macrocycles. The macrocycles above mainly consisted of imidazole analogs and aromatic fragments. Šindelář and Lizal [36] also presented the synthesis of hybrid macrocycles containing glycoluril and aromatic units. In 2014, Keinan et al. [37] reported a series of macrocycles, consisting of alternating urea or thiourea and phenol units, namely, multifarenes. Hitherto, multifarenes and their derivatives have been applied as fluorescence and electrochemical chemosensors [38–45].

Herein, we wish to report our endeavor in the facile synthesis of a new hemicucurbituril homologue, aminobenzene-containing hemicucurbituril **4** (Scheme 1). It was assumed that the amino groups could act as reactive sites for derivatization, at the same time allowing for formation of coordination or hydrogen bonds with guests, and the aminobenzene unit as a chromophore could improve the optical properties. With this novel macrocycle in hand, the interactions with some metal cations have been initially studied. Among the metal cations examined, the fluorescence intensity of macrocycle **4** quenched significantly when adding the corresponding equivalents of Fe^3+^ and Cu^2+^. Notably, this macrocyclic host molecule formed 1:1 complexes with Fe^3+^ in DMF with an association constant up to (2.1 ± 0.3) × 10^4^ M^−1^, which was contrary to those of hemicucurbiturils. As observed by fluorescence titration experiments, macrocycle **4** showed weak interactions with selected anions.

## Results and Discussion

**Scheme 2:**
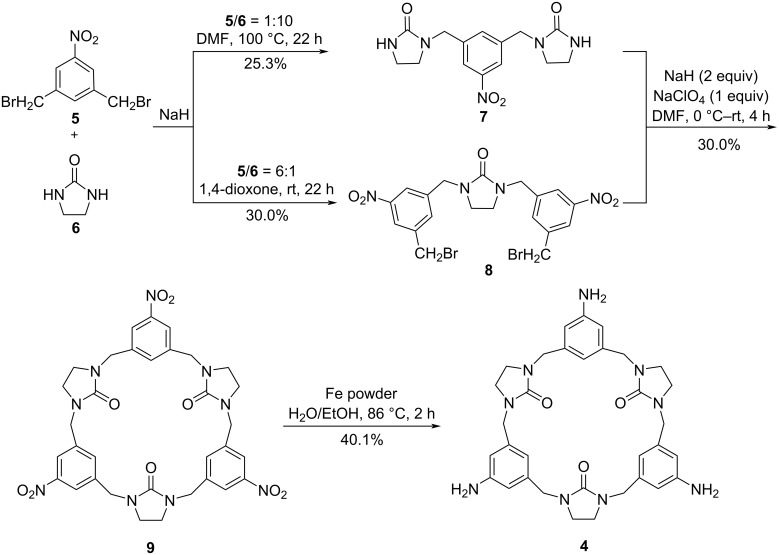
The route for the synthesis of aminobenzene-containing hemicucurbituril **4**.

Because of the relatively strong nucleophilicity of the amino group in aniline, the synthesis could become complicated when aminobenzene is directly used as the starting substrate. Thus, the study was commenced with the synthesis of the nitrobenzene-containing hemicucurbituril **9** based on a fragment coupling strategy (Scheme 2). For this purpose, 1,3-bis(bromomethyl)-5-nitrobenzene, which was easily obtained by reduction of 5-nitroisophthalic acid with NaBH_4_ and BF_3_·Et_2_O followed by subsequent bromination with PBr_3_ [46] and 2-imidazolidinone (**6**) were used as building blocks. By controlling the molar ratio of **5** and **6** at 1:10 or 6:1 and the reaction conditions, products **7** and **8** were readily accessible with 25.3% yield and 30.0% yield based on starting compounds **5** and **6**, respectively (Scheme 2). With the trimers **7** and **8** in hand, the reaction conditions for the [3 + 3] macrocyclic condensation were examined.

Due to the low solubility of compound **7**, the subsequent reactions were conducted in DMF. With two equivalents of Cs_2_CO_3_ as the base and with or without an additive (Table 1, entries 1–4), no desired product was isolated. When the base was changed to NaH, to our delight, the macrocycle **9** was obtained even without any additive with 26.0% yield (Table 1, entry 5). As is known that the template plays an important role in the cyclization, some additives were examined in combination with NaH as the base for improving the yield (Table 1, entries 6–9). For the synthesis of hemicucurbituril derivatives in a previous study, halide ions usually facilitated the cyclization [27]. However, halide ions had no effect on the current process (Table 1, entries 6–8). This observation could infer that the nitrobenzene-containing hemicucurbituril **9** shows no obvious affinity to halide ions such as chloride and bromide. When NaClO_4_ was introduced as the additive, the yield was moderately improved to 30.0% (Table 1, entry 9). Prolonging the reaction time showed no obvious effect on the reaction (Table 1, entry 10).

**Table 1:** Studies on the reaction conditions for the synthesis of nitrobenzene-containing hemicucurbituril **9**.

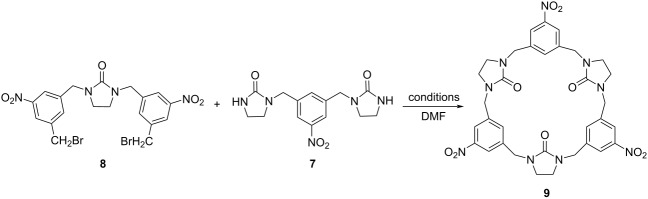					

Entry	Base (equiv)	Additive (equiv)	Time (h)	Temperature (°C)	Yield^a^ (%)

1^b^	Cs_2_CO_3_ (2)	–	24	100	–
2^b^	Cs_2_CO_3_ (2)	TBACl (2)	24	100	–
3^b^	Cs_2_CO_3_ (2)	TBABr (2)	24	100	–
4^b^	Cs_2_CO_3_ (2)	NaI (2)	24	100	–
5^c^	NaH (2)	–	4	0–rt	26.0
6^c^	NaH (2)	TBACl (1)	4	0–rt	25.0
7^c^	NaH (2)	TBABr (1)	4	0–rt	25.2
8^c^	NaH (2)	NaI (1)	4	0–rt	complex mixture
**9** **^c^**	**NaH (2)**	**NaClO** **_4_** ** (1)**	**4**	**0–rt**	**30.0**
10^c^	NaH (2)	NaClO_4_ (1)	12	0–rt	29.2

^a^Isolated yield based on **7**; ^b^reactions were carried out with **7** (2.2 mmol), **8** (2.2 mmol) and Cs_2_CO_3_ (4.4 mmol) in DMF (150 mL); ^c^NaH (4.4 mmol) was added to a solution of **7** (2.2 mmol) in DMF (150 mL) at 0 °C. After stirring for 5 min, the corresponding additive and **8** (2.2 mmol) were subsequently added, and then the mixture was stirred at rt.

**Figure 1:**
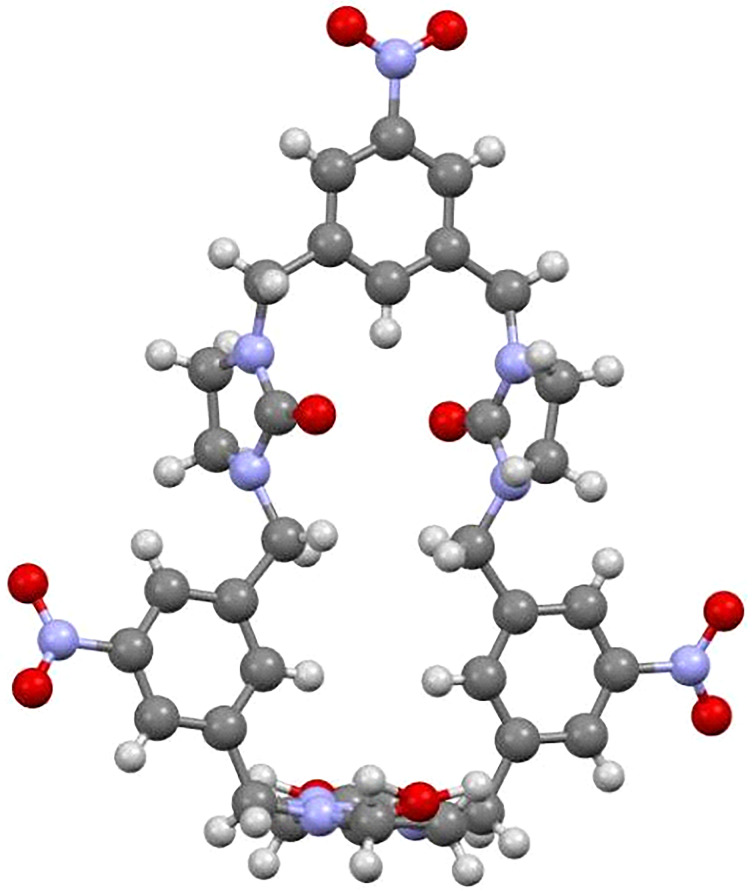
The X-ray structure of nitrobenzene-containing hemicucurbituril **9** (CCDC 2094879).

Macrocycle **9** was obtained as a yellowish solid and dissolving it in a mixture of dichloromethane and methanol, a yellowish crystal was obtained by gradually evaporating the solvent, which was suitable for X-ray diffraction analysis. As shown in Figure 1, the nitrobenzene-containing hemicucurbituril **9** adopted a square-cavity conformation. Notably, three nitrobenzene units shared nearly a plane. It should be highlighted that macrocycle **9** gave concise proton and carbon signals in the ^1^H and ^13^C NMR spectra, respectively. As displayed in Figure S6 (Supporting Information File 1), only two sets of proton peaks and four sets of carbon peaks corresponding to aromatic portions of the aforementioned macrocycle, in addition to two sets of proton peaks corresponding to bridged methylene and methylene of imidazolidinone components were observed at rt. This indicated that the formed macrocycle is highly symmetric in solution.

After successful construction of macrocycle **9**, the aminobenzene-containing hemicucurbituril **4** was obtained by reduction of the nitro groups in **9** with Fe powder in H_2_O/EtOH at 86 °C for 2 h with 40.1% yield (Scheme 2). Similar to the macrocyle **9**, the NMR spectra of aminobenzene-containing hemicucurbituril **4** (Figure S7, Supporting Information File 1) revealed that the compound is also highly symmetric. It is worth mentioning, that the macrocycle **4** is the first example of an aminobenzene-containing hemicucurbituril analog.

**Figure 2:**
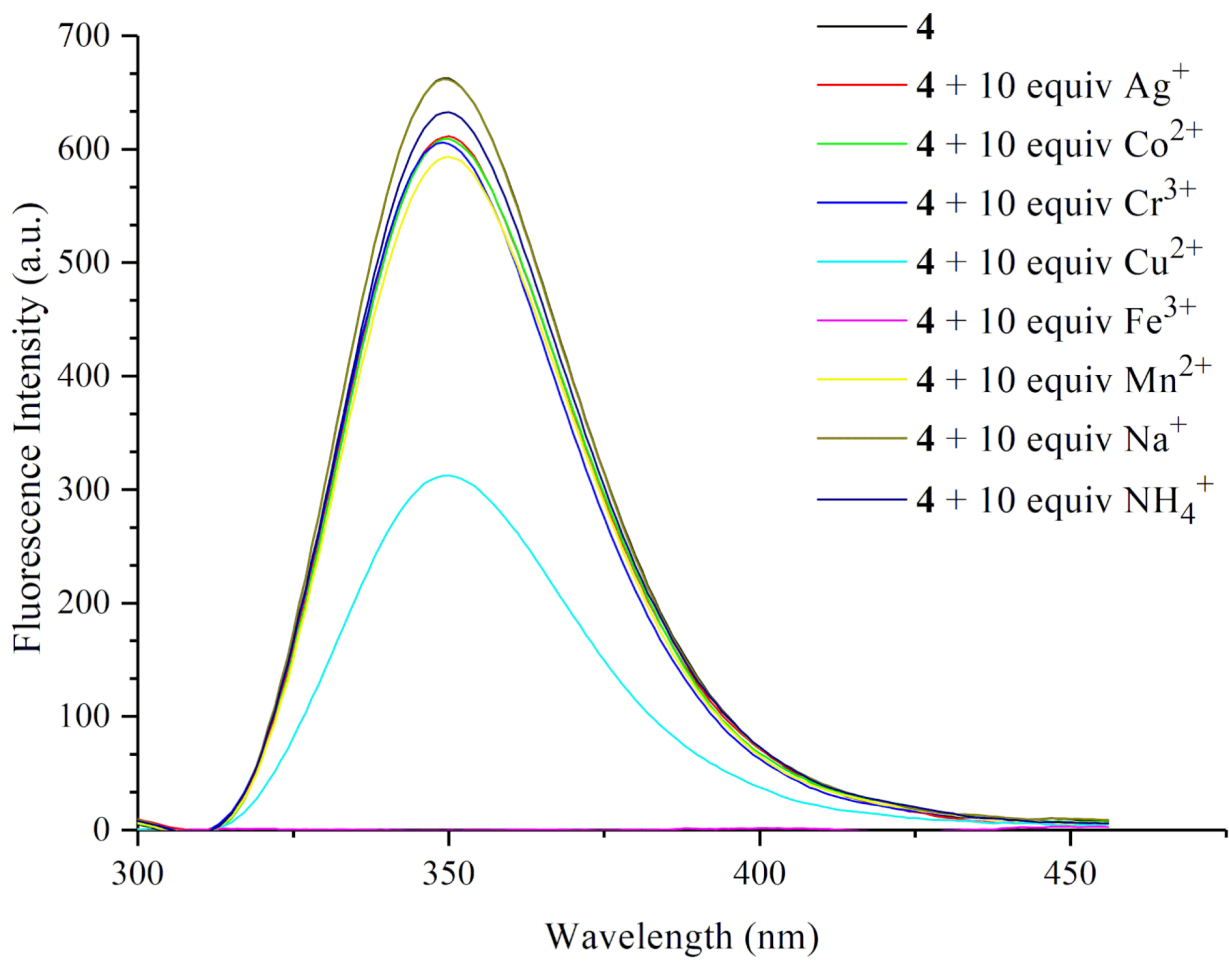
Fluorescence emission spectra (λ_max_ = 349 nm) of **4** (2.5 × 10^−5^ M) in the presence of 10 equivalents of Ag^+^, Co^2+^, Cr^3+^, Cu^2+^, Fe^3+^, Mn^2+^, Na^+^, NH_4_^+^ (2.5 × 10^−4^ M) in DMF at 298 K.

**Figure 3:**
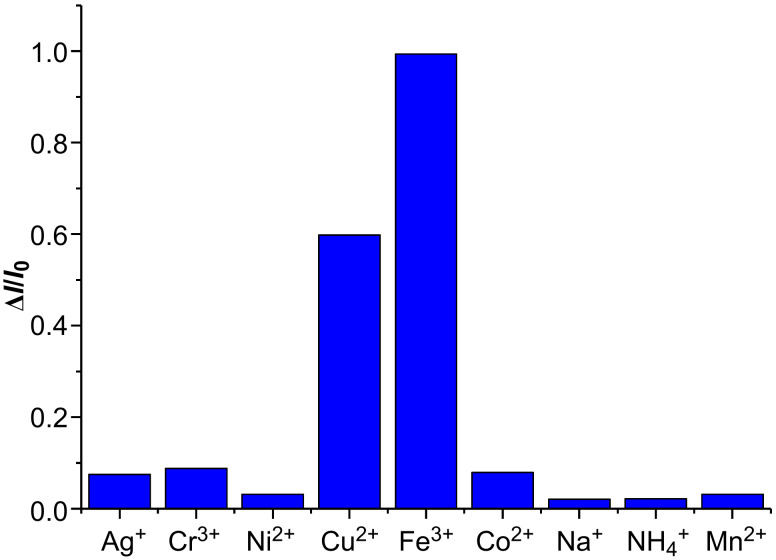
Column diagram of fluorescence quenching efficiency of **4** (2.5 × 10^−5^ M) in the presence of 10 equivalents of Ag^+^, Cr^3+^, Ni^2+^, Cu^2+^, Fe^3+^, Co^2+^, Na^+^, NH_4_^+^ and Mn^2+^ (2.5 × 10^−4^ M) in DMF at 298 K (nitrate salts were used as cation source).

**Figure 4:**
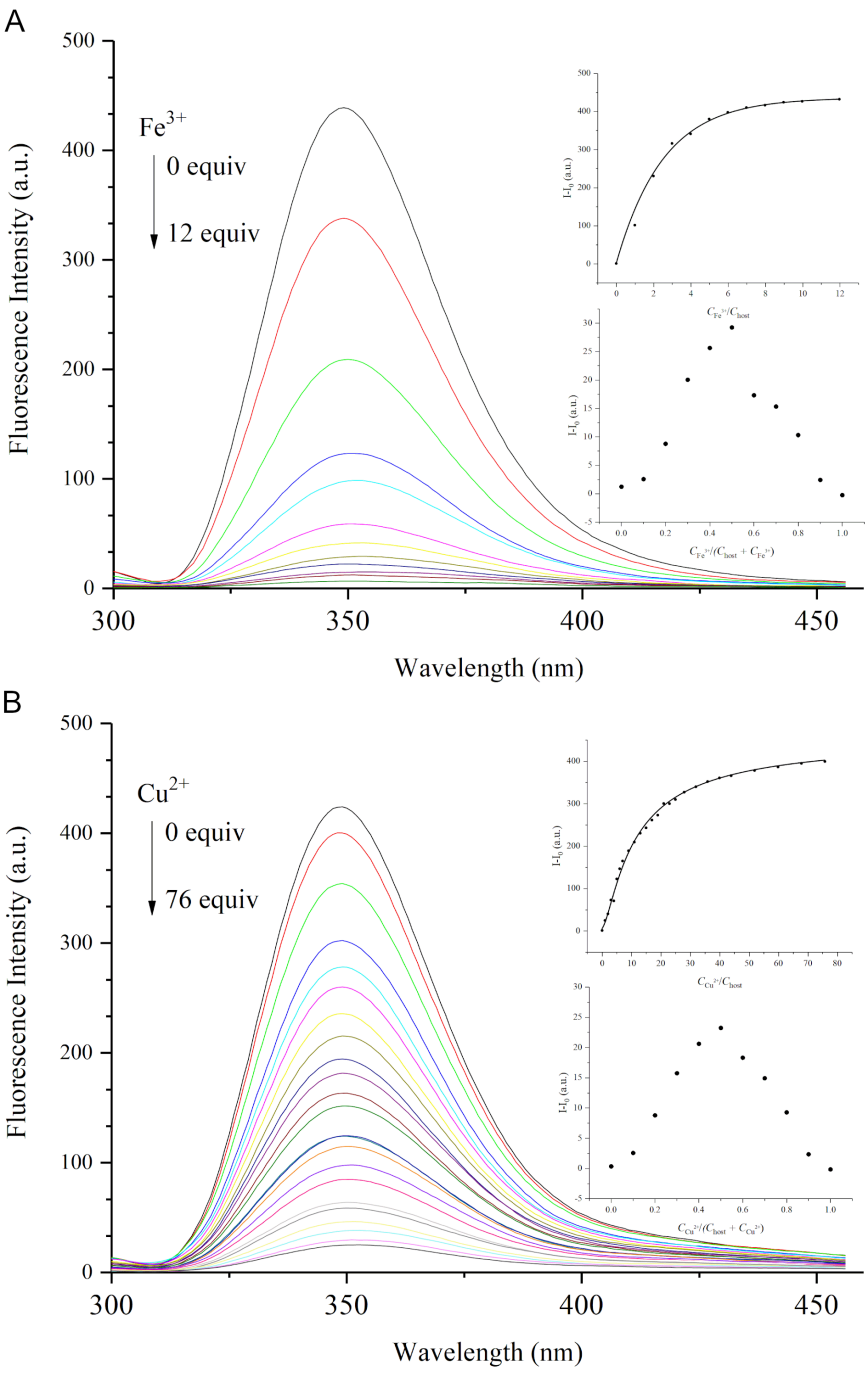
Fluorescence emission spectra (λ_max_ = 349 nm) of **4** (2.5 × 10^−5^ M) in the presence of Fe^3+^ and Cu^2+^ in DMF at 298 K, respectively. A) The concentration of Fe^3+^ for curves from top to bottom were 0.0, 2.5, 5.0, 7.5, 10.0, 12.5, 15.0, 17.5, 20.0, 22.5, 25.0, 27.5 (× 10^−5^ M) with increasing Fe^3+^ concentration. B) The concentration of Cu^2+^ for curves from top to bottom were 0.0, 10.0, 20.0, 30.0, 40.0, 50.0, 60.0, …, 190.0 (× 10^−5^ M) with increasing Cu^2+^ concentration. Insets: The inset on top is the nonlinear fitting curve of the fluorescence intensity *I − I*_0_ with increasing Fe^3+^ or Cu^2+^ concentration. The bottom inset is the Job’s plot for the **4**–Fe^3+^ or **4**–Cu^2+^ complex in DMF solution. ([4] + [Fe^3+^] = 2.5 × 10^−5^ M, [4] + [Cu^2+^] = 2.5 × 10^−5^ M).

With the novel macrocycle **4** in hand, initial experiments were conducted to explore its properties on recognizing metal cations. The fluorescence titration of macrocycle **4** with a series of selected cations (Figure 2), including Ag^+^, Na^+^, NH_4_^+^, Cu^2+^, Co^2+^, Cr^3+^, Fe^3+^, Ni^2+^, and Mn^2+^ (nitrate salts were used as cation sources) in DMF resulted in different degrees of quenching of the fluorescence emission of host **4**. The results are collected in Figure 3 as the corresponding fluorescence quenching efficiency which was quantified using the equation Δ*I = (I*_0 _*− I)*, where *I* is the fluorescence intensity response of the aminobenzene-containing hemicucurbituril **4** in the presence of metal cations, and *I*_0_ represents the corresponding fluorescence intensity in the absence of such metal cations. The addition of Fe^3+^ and Cu^2+^ caused a significant quenching of the fluorescence intensity. Evidenced by the fluorescence titration and Job’s plot experiments (Figure 4, bottom inset), aminobenzene-containing hemicucurbituril **4** interacts with Fe^3+^ and Cu^2+^ by forming a 1:1 complex in DMF solution. After depicting non-linear fitting curve and based on the titration data, the association constants for 1:1 complexation between the host **4** and the guest cations were calculated by Equation 1 [47],


[1] $$\Delta I=\frac{\Delta a\left({\left[H\right]}_{0}+{\left[G\right]}_{0}+\frac{1}{K_{a}}\right)\pm \sqrt{\Delta a^{2}{\left({\left[H\right]}_{0}+{\left[G\right]}_{0}+\frac{1}{K_{a}}\right)}^{2}-4\Delta a^{2}{\left[H\right]}_{0}{\left[G\right]}_{0}}}{2}$$

where *K*_a_ is the association constant of the host–guest interaction, while Δ*I* is the change in the fluorescence intensity of the host upon gradual addition of the guest, and Δa refers to the different constant between the free guest and the interaction complex; the initial concentrations of host and guest are denoted by [H]_0_ and [G]_0_, respectively. As summarized, the association constant of aminobenzene-containing hemicucurbituril **4** with Fe^3+^ was obtained from the change in fluorescence intensity at 349 nm, and found to be *K*_a_ = (2.1 ± 0.3) × 10^4^ M^−1^, while the association constant of compound **4** binding to Cu^2+^ was *K*_a_ = (2.8 ± 0.1) × 10^3^ M^−1^. The macrocycle binds Fe^3+^ much more strongly than Cu^2+^.

**Figure 5:**
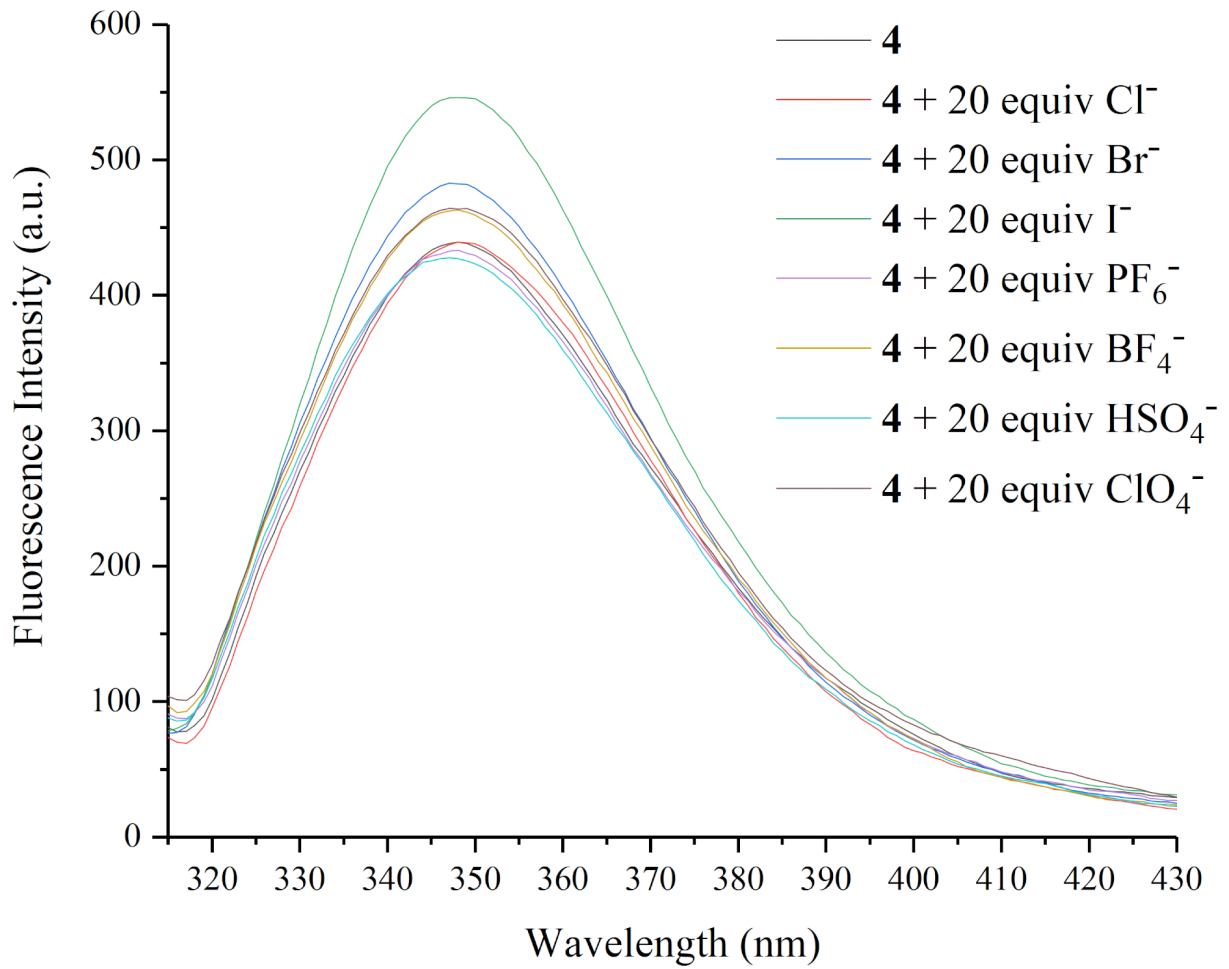
Fluorescence emission spectra (λ_max_ = 349 nm) of **4** (2.5 × 10^−5^ M) in the presence of 20 equivalents of Cl^−^, Br^−^, I^−^, PF_6_^−^, BF_4_^−^, HSO_4_^−^, ClO_4_^−^ (5 × 10^−4^ M) in CH_2_Cl_2_/CH_3_OH 4:1 (v/v) at 298 K.

**Figure 6:**
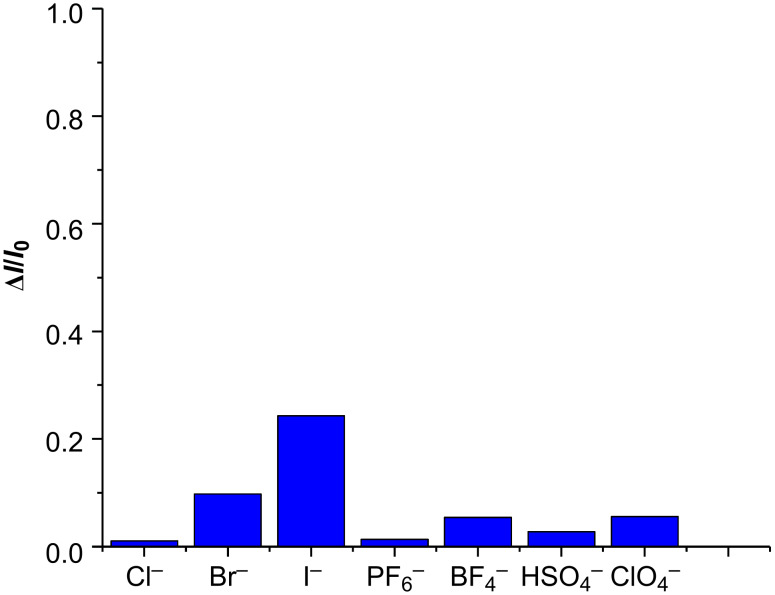
Column diagram of fluorescence enhancement efficiency of **4** (2.5 × 10^−5^ M) in the presence of 20 equivalents of Cl^−^, Br^−^, I^−^, PF_6_^−^, BF_4_^−^, HSO_4_^−^, and ClO_4_^−^ (5.0 × 10^−4^ M) in CH_2_Cl_2_/CH_3_OH 4:1 (v/v) at 298 K (tetrabutylammonium salts were used as anion source).

The host–guest interactions between **4** and selected anions, including Cl^−^, Br^−^, I^−^, PF_6_^−^, BF_4_^−^, HSO_4_^−^, and ClO_4_^−^ (tetrabutylammonium salts were used as anion source) were also tested tentatively by fluorescence titration (Figure 5). The overwhelming majority of aromatic molecules follow the heavy-atom effect rule [48]. However, it was surprisingly found that the coordination of selected anions, such as halide ions, especially the heavier iodide ion, to the macrocyclic sensor, slightly enhanced the fluorescence emission in CH_2_Cl_2_/CH_3_OH 4:1 (v/v) at 298 K, instead of quenching the fluorescence as predicted by the classic heavy-atom effect. The corresponding fluorescence enhancement efficiency of selected anions is collected in Figure 6. The addition of 20 equivalents of I^−^ ions resulted in an approximately 24% enhancement of the fluorescence intensity, while other anions induced either no obvious change (Cl^−^, PF_6_^−^, and HSO_4_^−^) or only a slight enhancement (Br^−^, BF_4_^−^, and ClO_4_^−^) of the fluorescence intensity.

## Conclusion

In summary, we have presented a novel type of hemicucurbituril derivative modified with aminobenzene. Based on the fragment coupling strategy, the nitrobenzene-containing hemicucurbituril **9** was synthesized by nucleophilic substitution reaction with two fragments **7** and **8**. The desired aminobenzene-containing hemicucurbituril **4** was achieved by reduction of the corresponding nitrobenzene-containing hemicucurbituril **9**. In the initial host–guest study, this novel macroycle **4** exhibited strong interaction with Fe^3+^ and formed a 1:1 complex with the association constant of *K*_a_ = (2.1 ± 0.3) × 10^4^ M^−1^. In another perspective, the presence of the coordination of selected anions to the macrocyclic sensor enhanced the fluorescence emission in various degree, extremely contrary to the classic heavy-atom effect caused by a heavy atom. In general, this macrocyclic sensor showed high fluorescence quenching efficiency toward Fe^3+^ and Cu^2+^ over other cations and generally low fluorescence enhanced efficiency with selected anions.

Overall, the fluorescence response properties of aminobenzene-containing hemicucurbituril and its readily modifiable nature, provide a convenient platform for the exploration on host–guest interaction and supramolecular systems. Its applications and modifications are being pursued in our laboratory, and the results will be reported in due course.

## Supporting Information

File 1 Experimental procedures, characterization data and copies of spectra.
