# Supplementary material for: Synthesis of a novel aminobenzene-containing hemicucurbituril and its fluorescence spectral properties with ions
Source: Beilstein J Org Chem. 2021 Dec 6;17:2840–7. doi: 10.3762/bjoc.17.195 (PMC8685562; doi:10.3762/bjoc.17.195)
Supplement: File 1 — Experimental procedures, characterization data and copies of spectra. [file Beilstein_J_Org_Chem-17-2840-s001.pdf]

BEILSTEIN JOURNAL OF ORGANIC CHEMISTRY

Supporting Information

for

Synthesis of a novel aminobenzene-containing hemicucurbituril and its fluorescence spectral properties with ions

Qingkai Zeng, Qiumeng Long, Jihong Lu, Li Wang, Yuting You, Xiaoting Yuan, Qianjun Zhang, Qingmei Ge, Hang Cong and Mao Liu

Beilstein J. Org. Chem. **2021,** *17,* 2840–2847. doi:10.3762/bjoc.17.195

Experimental procedures, characterization data and copies of spectra

License and Terms: This is a supporting information file under the terms of the Creative Commons Attribution License (https://creativecommons.org/licenses/by/4.0). Please note that the reuse, redistribution and reproduction in particular requires that the author(s) and source are credited and that individual graphics may be subject to special legal provisions.
The license is subject to the *Beilstein Journal of Organic Chemistry* terms and conditions: (https://www.beilstein-journals.org/bjoc/terms)

Table of contents

1. General information S2
2. Experimental procedures and characterization of products S3
3. UV−vis absorption and fluorescence spectroscopy data S5
4. Crystal data and structure refinement for X-ray structure S6
5. Copies of ^{1}H and ^{13}C NMR spectra S9

1. General information

Apparatus and materials

All commercially available reagents were used as received. TLC analysis was performed on pre-coated, glass-backed silica gel plates and visualized with UV light. Flash column chromatography was performed on silica gel (200–300 mesh). Anhydrous DMF was dried over 4 Å molecule sieves. ^{1}H NMR spectra were recorded on a 400 MHz NMR spectrometer (JEOL JNMECZ-400) and ^{13}C NMR spectra were recorded on a 100 MHz NMR spectrometer (JEOL JNMECZ-400). UV–vis absorption spectra were recorded on a Unico UV-2102 instrument at 25 °C. Fluorescence spectra were recorded on a Varian RF-540 fluorescence spectrophotometer at 25 °C. Mass spectra were recorded on an Agilent 6545 Q-TOF LC/MS instrument. According to reference[1], compound **5** was synthesized by reduction of 5-nitroisophthalic acid with $NaBH_4$ and BF_3, followed by bromination with PBr_3。

Fluorescence titration of the host–guest complexes

The solution of the host **4** was prepared at a fixed concentration of 2.5×10^{-5} mol·L^{-1} in DMF. Parts of this solution were combined with metal cations in C_{host}: C_{guest} ratios of 1:1, 1:2, 1:3, 1:4, 1:5, 1:6, 1:7, 1:8 …, and 1:80. Another set of solutions was prepared at a fixed concentration of 2.5×10^{-5} mol·L^{-1} in CH_2Cl_2/CH_3OH 4:1 (v/v) and combined with selected anions in C_{host}: C_{guest} ratios of 1:1, 1:2, 1:3, 1:4, 1:5, 1:6, 1:7, 1:8 …, and 1:20. For the Job plots, the total concentration of ($C_{\text{host}} + C_{\text{guest}}$) was 2.5×10^{-5} mol·L^{-1}. For aminobenzene-containing hemicucurbituril **4**, the emission wavelength was λ_{em} = 349 nm upon excitation at λ_{ex} = 294 nm. The association constants of the host–guest interactions were calculated by nonlinear fitting employing the equation (1) [2].

References

[1] Chhikara, B. S.;Kumar, N.;Tandon, V.,Mishra, A. K. Biorg. Med. Chem. **2005**, 13, 4713-4720.
[2] Thordarson, P. Chem. Soc. Rev. **2011**, 40, 1305-1323.

2. Experimental procedures and characterization of products.

Synthesis of 7. 2-Imidazolidione (**6**, 174.24 mmol, 15.00 g) was completely dissolved in anhydrous DMF (45 mL) in a round-bottomed flask. Then, compound **5** (16.18 mmol, 5.00 g) was added to the solution and the mixture stirred at 100 ℃ overnight. The reaction was quenched with distilled water (100 mL) and extracted with CH_2Cl_2 (3 × 100 mL). The combined organic layer was washed with brine (3 × 100 mL), dried over anhydrous Na_2SO_4, and concentrated under vacuum. The residue was purified by column chromatography on silica gel (200–300 mesh) using a mixture of ethyl acetate and methanol (v/v, 10:1) as eluent to give product **7** as a white solid, yield: 1.31 g, 25.3%.

7: ^{1}H NMR (400 MHz, Chloroform-*d*): δ = 8.05 (s, 2H), 7.57 (s, 1H), 4.46 (s, 4H), 3.56-3.43 (m, 4H), 3.43-3.31 (m, 4H); ^{13}C NMR (100 MHz, Chloroform -*d*): δ = 162.6, 148.9, 140.3, 133.3, 121.8, 47.3, 45.1, 38.2; HRMS ESI Calcul ated for $C_{14}H_{17}N_5O_4$ $[M+H]^+$: 320.1353, Found: 320.1349.

Synthesis of 8. 2-Imidazolidione (**6**, 11.62 mmol, 1.00 g) was completely dissolved in 1,4-dioxone (50 mL). Then, compound **5** (69.59 mmol, 21.50 g) and sodium hydride (60%, dissolved in mineral oil, 47.52 mmol, 1.90 g) were added and the reaction mixture was stirred at room temperature overnight. NH_4Cl (56.09 mmol, 3.00 g) was added to neutralize pH value of the solution. The mixture was quenched by distilled water (100 mL) and extracted with CH_2Cl_2 (2 × 50 mL). The combined organic layer was washed with brine (3 × 100 mL), dried over anhydrous Na_2SO_4, and concentrated under vacuum. The residue was purified by column chromatography over silica gel (200–300 mesh) using a mixture of dichloromethane and methanol (v/v, 6:1) as an eluent to give product **8** as a white solid, yield: 1.89 g, 30.0%.

8: ^{1}H NMR (400 MHz, Chloroform-*d*): δ = 8.20 (s, 2H), 8.08 (s, 2H), 7.68 (s, 2H); 4.54 (s, 8H); 3.32 (s, 4H); ^{13}C NMR (100 MHz, Chloroform-*d*) : δ = 160.8, 148.7, 140.5, 140.3, 134.5, 123.4, 122.6, 48.0, 42.6, 31.2; HRMS ESI Calculated for $C_{19}H_{18}Br_2N_4O_5$ $[M+Na]^+$: 562.9544, Found: 562.9524.

Synthesis of 9. Compound **7** (2.20 mmol, 0.70 g) was dissolved in dry DMF (150 mL). Then, sodium hydride (60%, dissolved in mineral oil, 4.40 mmol, 0.18 g) was added to the solution, the mixture was stirred at 0 °C for 5 min, then $NaClO_4$ (2.20 mmol, 0.27 g) was added into the mixture and the solution gradually became yellow. Compound **8** (2.20 mmol, 1.19 g) was added and reaction mixture was stirred at room temperature for 4 h. NH_4Cl (5.60 mmol, 0.30 g) was added into the solution to neutralize pH value of solution. The mixture was quenched by distilled water (100 mL) and extracted with CH_2Cl_2 (3 × 100 mL). The combined organic layer was washed with brine (3 × 100 mL), dried over anhydrous Na_2SO_4, and concentrated under vacuum. The residue was purified by column chromatography over silica gel (200–300 mesh) using a mixture of ethyl acetate and methanol (v/v, 10:1) as an eluent to give product **9** as a white solid, yield: 0.46 g, 30.0%.

9: ^{1}H NMR (400 MHz, Chloroform-*d*): δ = 8.03 (s, 6H), 7.64 (s, 3H), 4.52 (s, 12H), 3.35 (s, 12H); ^{13}C NMR (100 MHz, Chloroform-*d*): δ = 161.1, 148.6, 140.3, 132.8 , 121.7, 47.9, 42.8; HRMS ESI Calculated for $C_{33}H_{33}N_9O_9$ $[M+Na]^+$: 722.2301; Found: 722.2297.

Synthesis of 4. Compound **9** (0.60 mmol, 0.42 g), NH_4Cl (9.35 mmol, 0.50 g), and iron powder (8.95 mmol, 0.50 g) in EtOH/H_2O 5:1 (v/v, 30 mL) were stirred at 85 °C for 1 h. The mixture was filtered while hot and dichloromethane/methanol 3:1 (v/v, 100 mL) was added to the filtrate. Then, NaS (solid) was added and stirring continued at 85 °C for 30 min. The mixture was filtered, the filtrate collected and concentrated under vacuum. The residue was purified by column chromatography over silica gel (200–300 mesh) using a mixture of dichloromethane/methanol 8:1 (v/v) as an eluent to give product **4** as a white solid, yield: 0.14 g, 40.1%.

4: ^{1}H NMR (400 MHz, DMSO-d_6) : δ = 6.34 (s, 6H), 6.19 (s, 3H), 5.16 (s, 6H), 4.11 (s, 12H), 3.00 (s, 12H); ^{13}C NMR (100 MHz, Chloroform-*d*) : δ = 160.4, 149.3, 138.3, 114.1, 112.3, 47.6, 41.5; HRMS ESI Calculated for $C_{33}H_{39}N_9O_3$ $[M+H]^+$: 610.3249; Found: 610.3227.

3. UV–vis absorption and fluorescence spectroscopy data of 4.

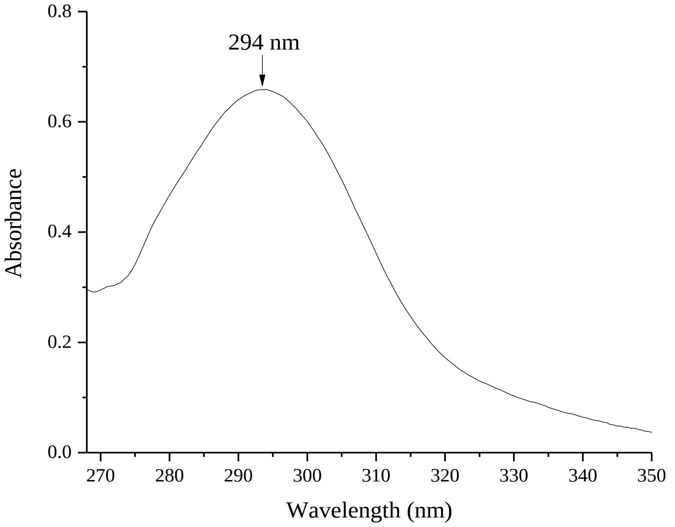

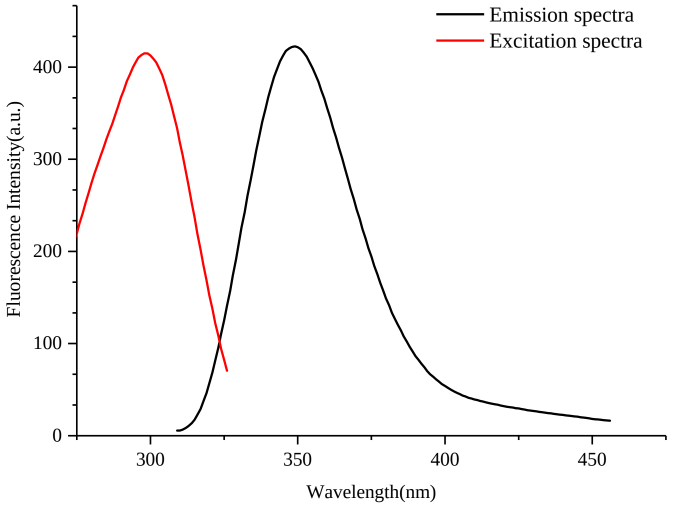

Figure S1: UV–vis absorption spectrum (top) of **4** (1.0×10^{-4} M) in DMF at 298 K and fluorescence excitation spectrum, emission spectrum (bottom) of **4** (2.5×10^{-5} M) in DMF at 298 K.

4. Crystal data and structure refinement for X-ray structure

Compound 9

Empirical formula	$C_{33}H_{33}N_9O_9$	
Formula weight	699.68	
Temperature	273.1500 K	
Crystal system	Orthorhombic	
Space group	Fddd	
Unit cell dimensions	a = 15.061(4) Å	α = 90 °
	b = 25.103(4) Å	β = 90 °
	c = 37.216(4) Å	γ = 90 °
Volume	14071(5) $Å^3$	
Z	16	
ρ_{calc}	1.321 g/cm^3	
μ	0.099 mm^{-1}	
F(000)	5856.0	
Radiation	MoKα (λ = 0.71073)	
2Θ range for data collection	3.914 ° to 56.044 °	
Index ranges	$-19 \leq h \leq 19, -32 \leq k \leq 32, -46 \leq l \leq 49$	
Reflections collected	105741	
Independent reflections	4256 [R_{int} = 0.1182, R_{sigma} = 0.0381]	
Data/restraints/parameters	4256 / 15 / 259	
Goodness-of-fit on F^2	1.099	
Final R indexes [I>=2σ (I)]	R_1 = 0.1065, wR_2 = 0.1934	
Largest diff. peak/hole	0.26 and -0.23 e $Å^{-3}$	

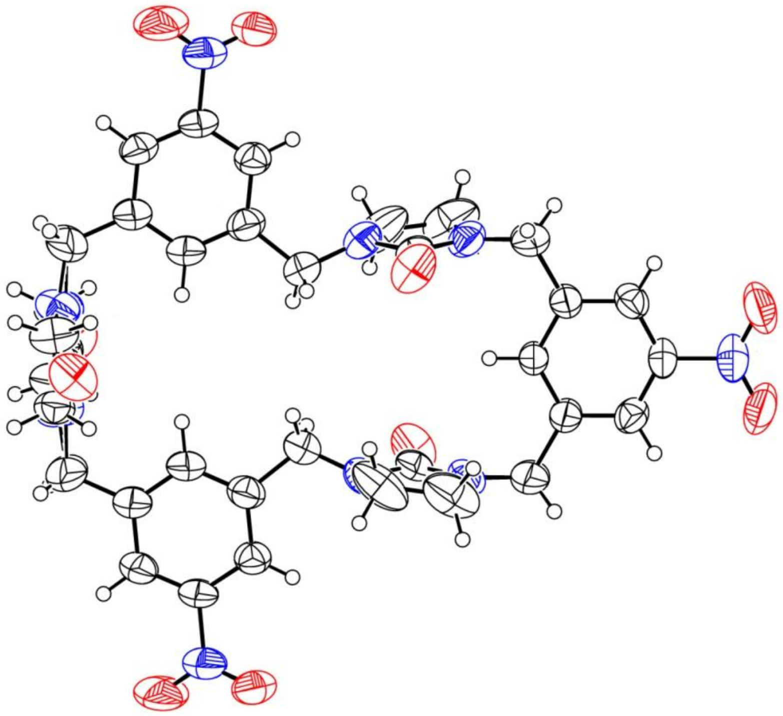

Figure S2: The X-ray structure of nitrobenzene-containing hemicucurbituril **9** (CCDC 2094879).

Atomic coordinates for X-ray structure

Fractional atomic coordinates ($\times10^4$) and equivalent isotropic displacement parameters ($Å^2\times10^3$) U (eq) is defined as 1/3 of of the trace of the orthogonalised U_{ij} tensor.

Atom	*x*	*y*	*z*	U(eq)
O1	5039(5)	6318(5)	5711(2)	86(2)
O2	6310(4)	9076.4(13)	5215.2(12)	152(2)
O3	5774(3)	8987.4(12)	4691.0(10)	86.6(10)
O4	7180(2)	7074.2(14)	3791.7(9)	85.2(10)
O5	6202(3)	5822.2(15)	1837.0(8)	104.5(13)
N1	6298(10)	5836(7)	5753(4)	58(3)
N2	6401(9)	6690(6)	5798(5)	72(4)
N3	6062(3)	8805.9(14)	4969.1(12)	76.8(11)
N4	5862(2)	7214.4(14)	4079.4(9)	65.2(9)
N5	5893(3)	7227.3(14)	3492.0(9)	68.7(10)
N6	6250	6250	1991.5(13)	76.9(15)
C1	6111(3)	8219.6(14)	5005.1(11)	57.5(10)
C2	6047(3)	7916.5(15)	4698.4(11)	58.0(10)
C3	6109(3)	7367.4(15)	4735.9(11)	62.1(11)
C4	6212(4)	7153.2(16)	5074.0(12)	75.2(13)
C5	6256(4)	7464.3(16)	5376.8(12)	72.2(12)
C6	6221(3)	8013.6(15)	5340.5(12)	65.9(11)
C7	6125(4)	6991.9(17)	4419.5(11)	87.4(16)
C8	4937(3)	7227(2)	3979.6(18)	107(2)
C9	4971(4)	7309(2)	3579.2(18)	111(2)
C10	6388(3)	7162.5(14)	3786.8(11)	53.9(9)
C11	6256(4)	7252.0(16)	3134.3(12)	89.3(16)
C12	6232(3)	6729.9(15)	2933.5(10)	58.1(10)
C13	6250	6250	3114.5(13)	58.8(14)
C14	6239(3)	6731.0(15)	2561.8(10)	57.2(10)
C15	6250	6250	2386.8(13)	54.0(13)
C16	6372(5)	7226.2(18)	5746.7(14)	114(2)
C17	7314(7)	6523(4)	5819(3)	72(3)
C18	7264(8)	5910(4)	5816(3)	69(3)
C19	5829(6)	6284(4)	5748(2)	58.0(19)

5. Copies of ^1H and ^{13}C NMR spectra.

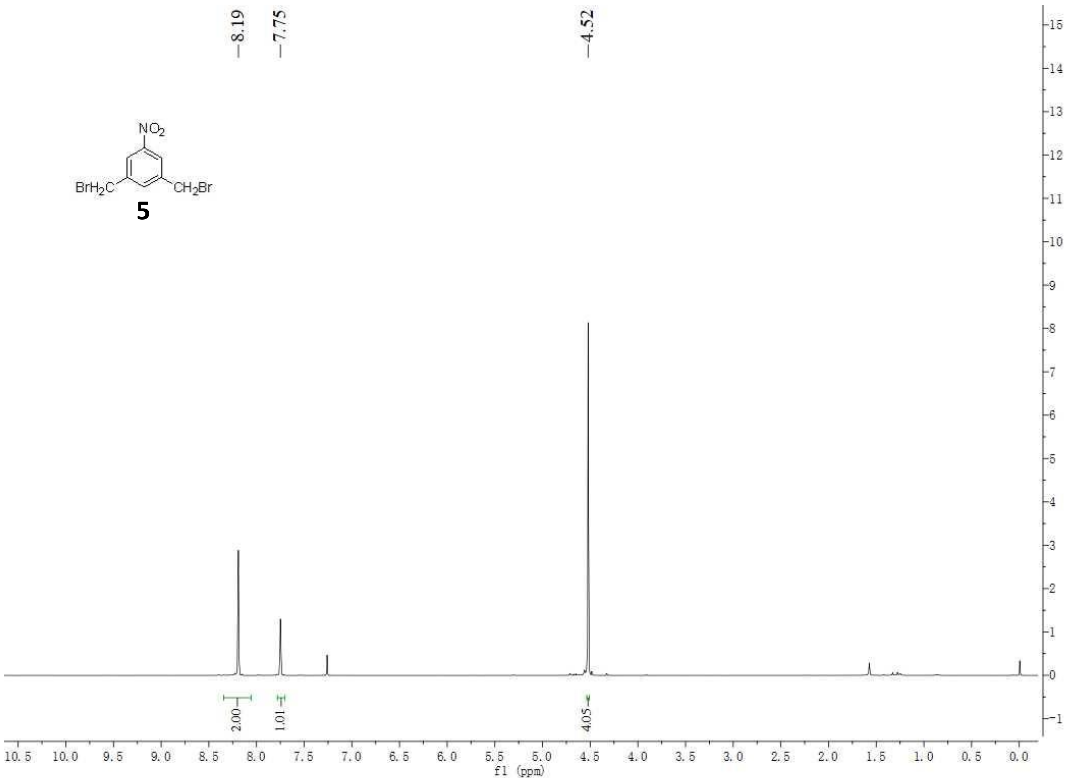

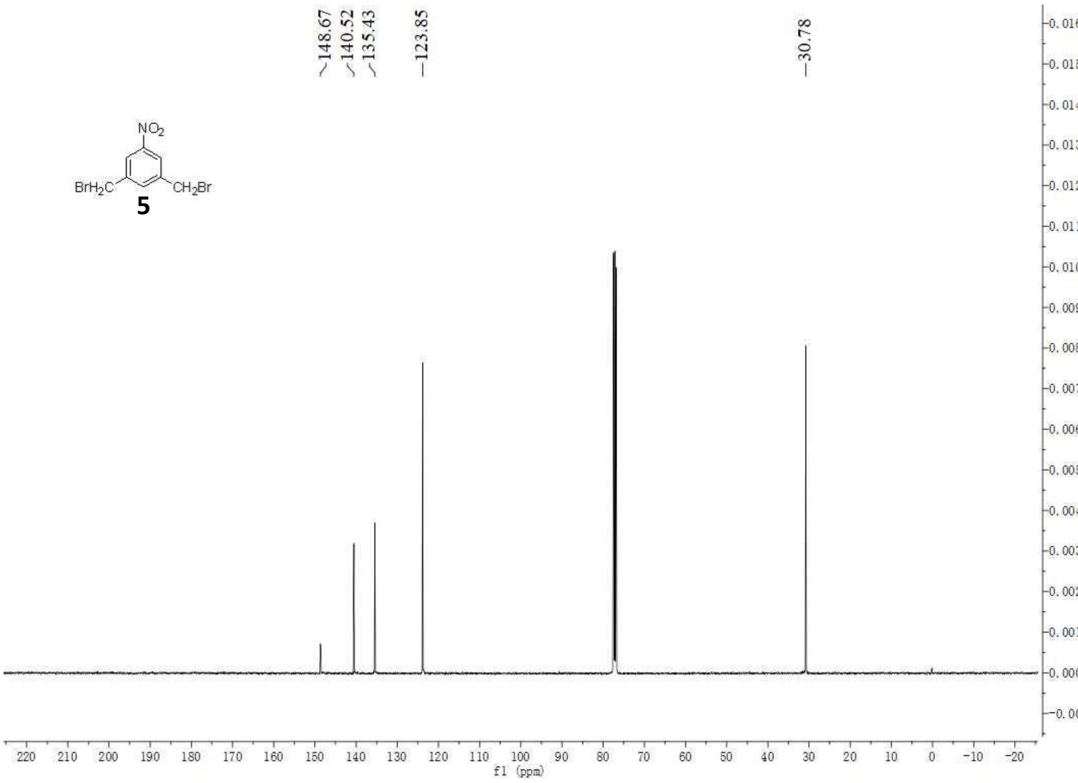

Figure S3: ^1H NMR spectrum (400 MHz, $CDCl_3$) and ^{13}C NMR spectrum (100 MHz, $CDCl_3$) of **5**.

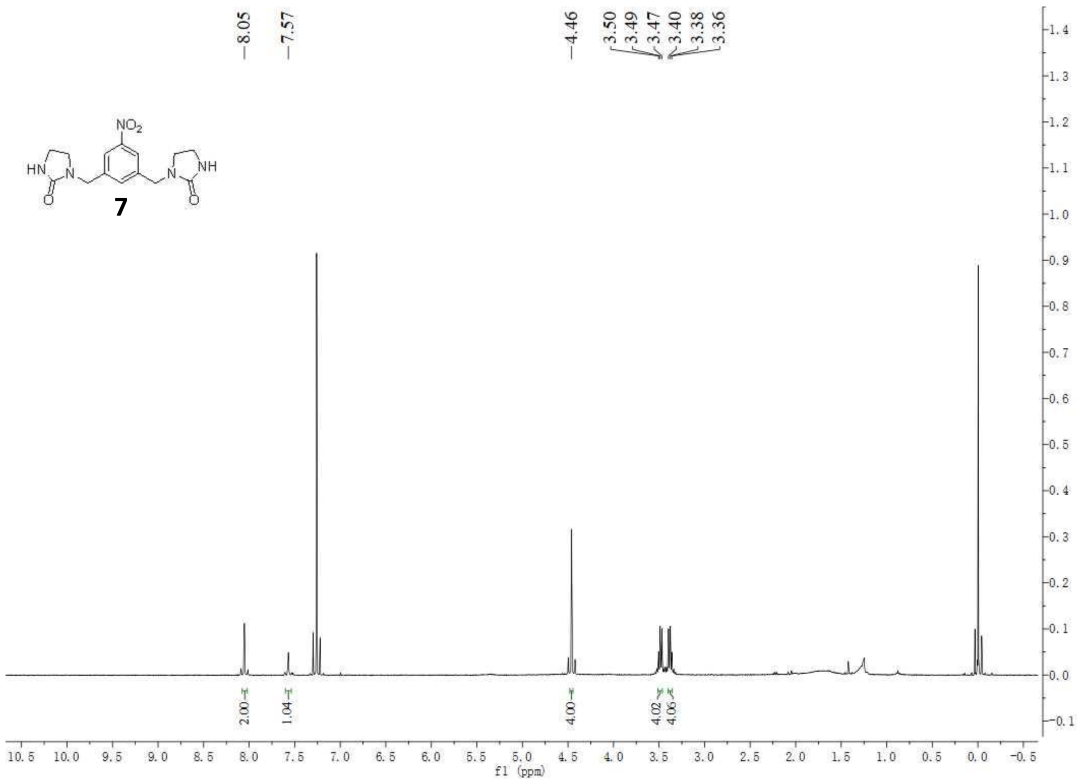

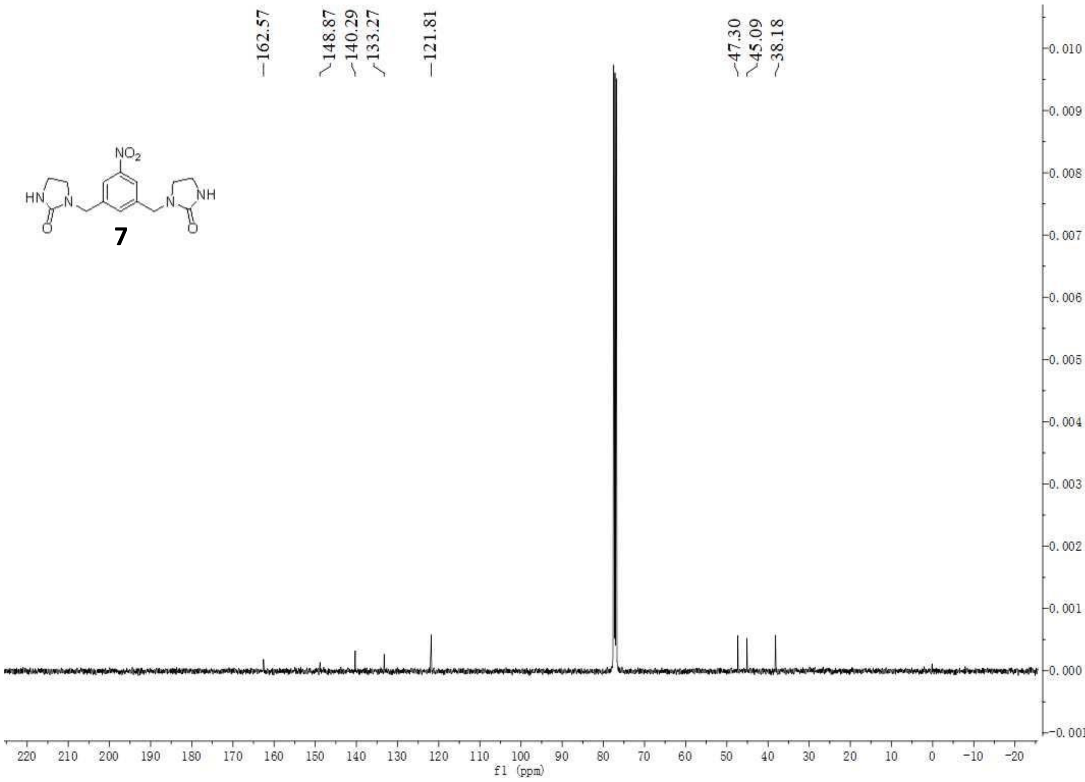

Figure S4: ^{1}H NMR spectrum (400 MHz, $CDCl_3$) and ^{13}C NMR spectrum (100 MHz, $CDCl_3$) of trimer **7**.

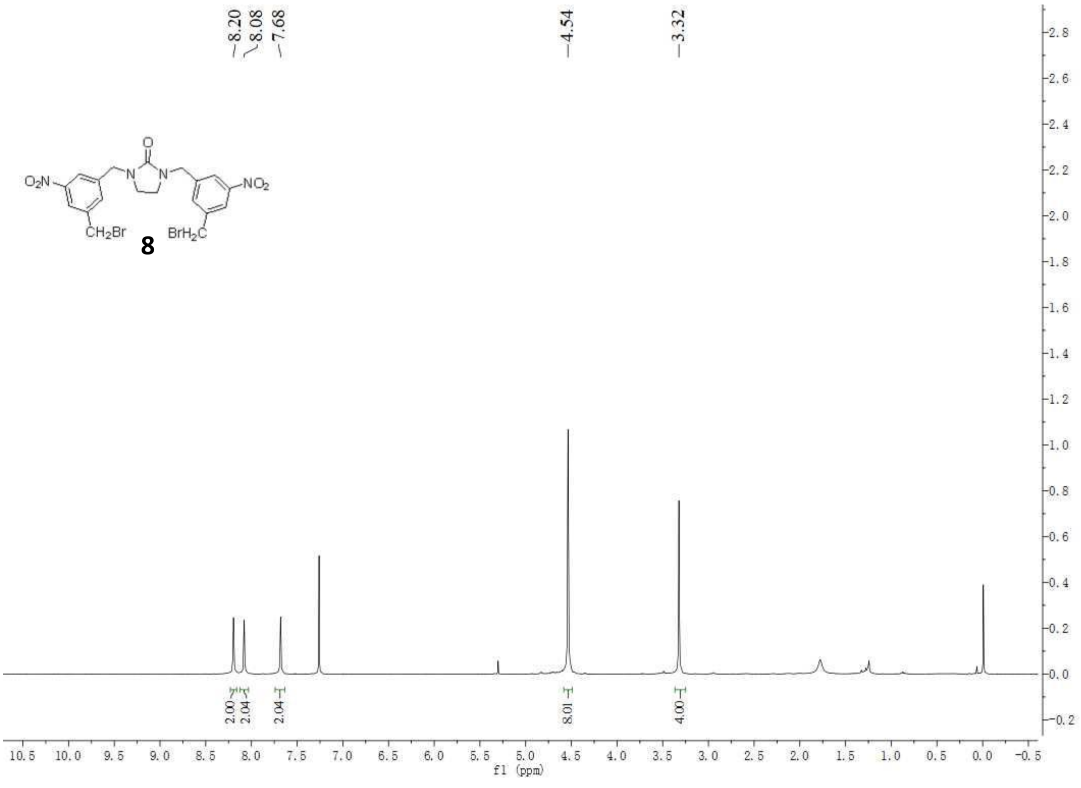

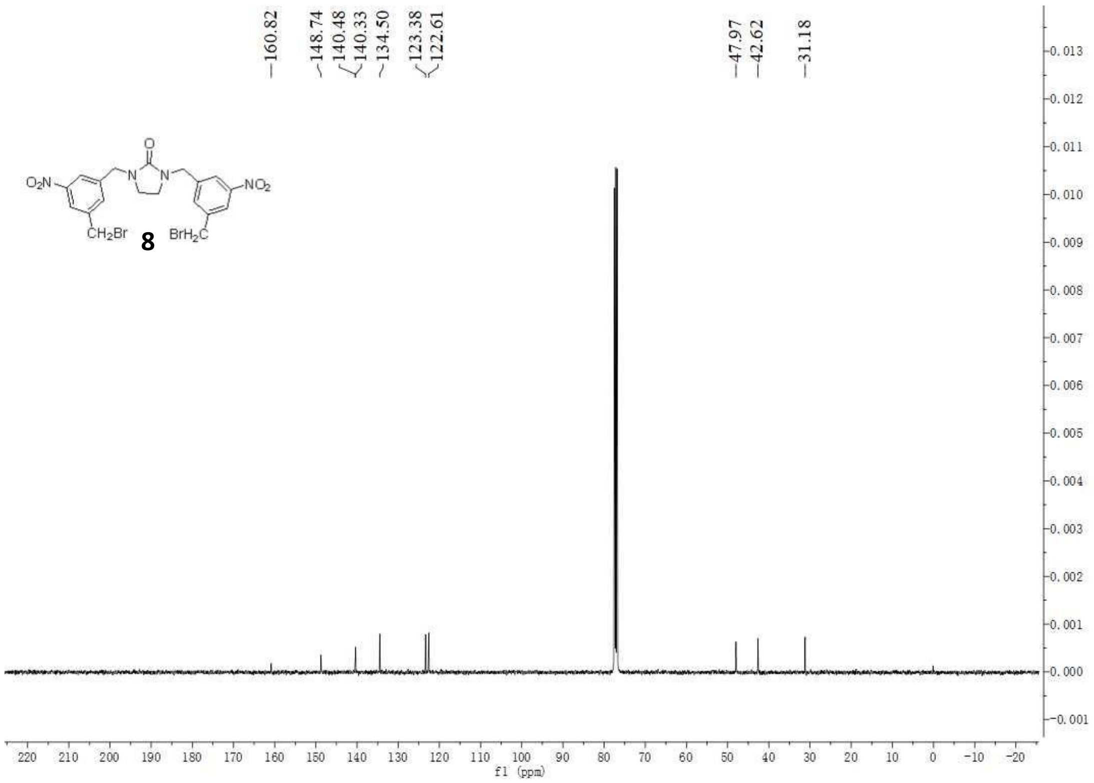

Figure S5: ^{1}H NMR spectrum (400 MHz, $CDCl_3$) and ^{13}C NMR spectrum (100 MHz, $CDCl_3$) of trimer **8**.

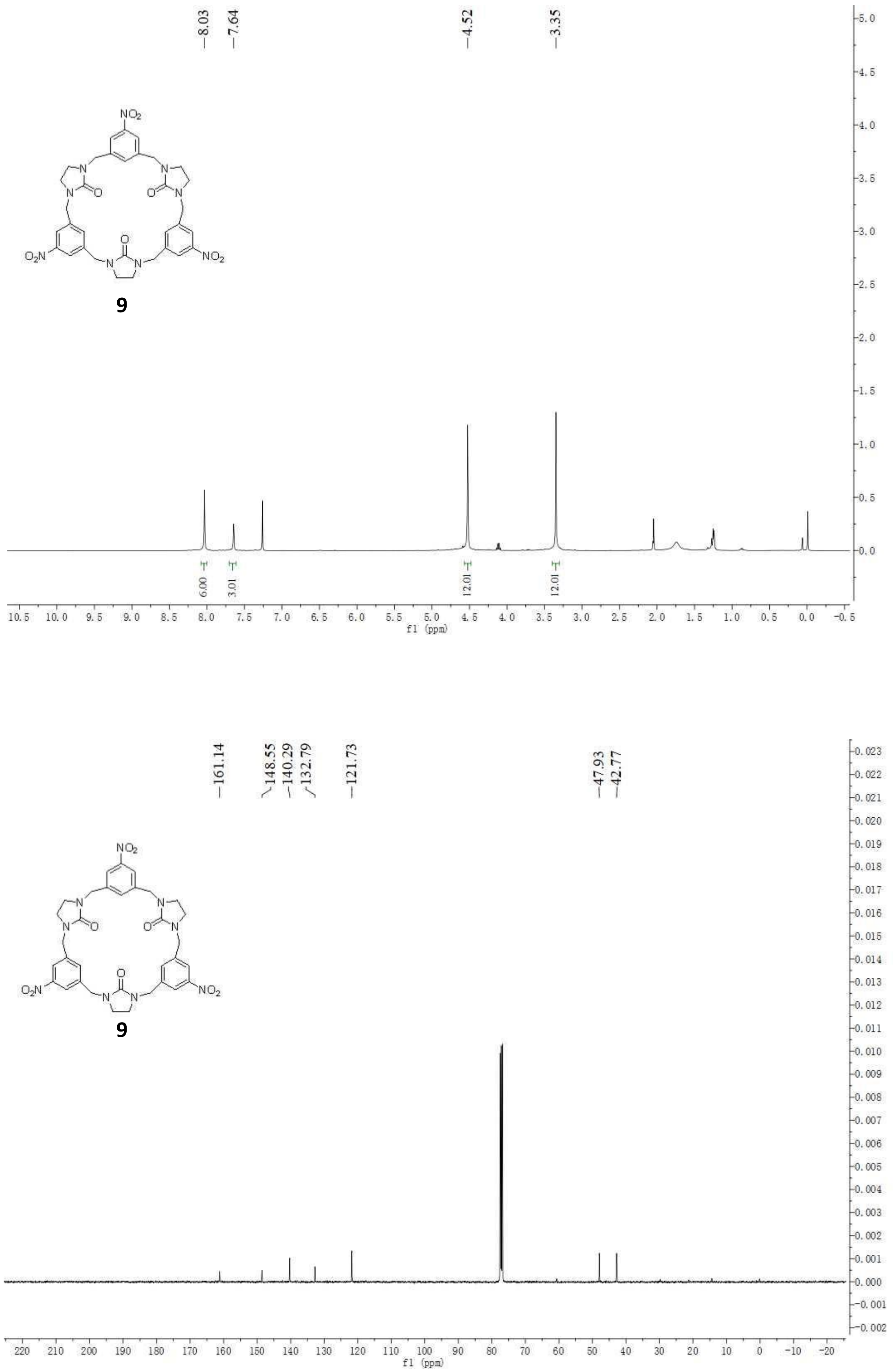

Figure S6: ^{1}H NMR spectrum (400 MHz, $CDCl_3$) and ^{13}C NMR spectrum (100 MHz, $CDCl_3$) of nitrobenzene-containing hemicucurbituril **9**.

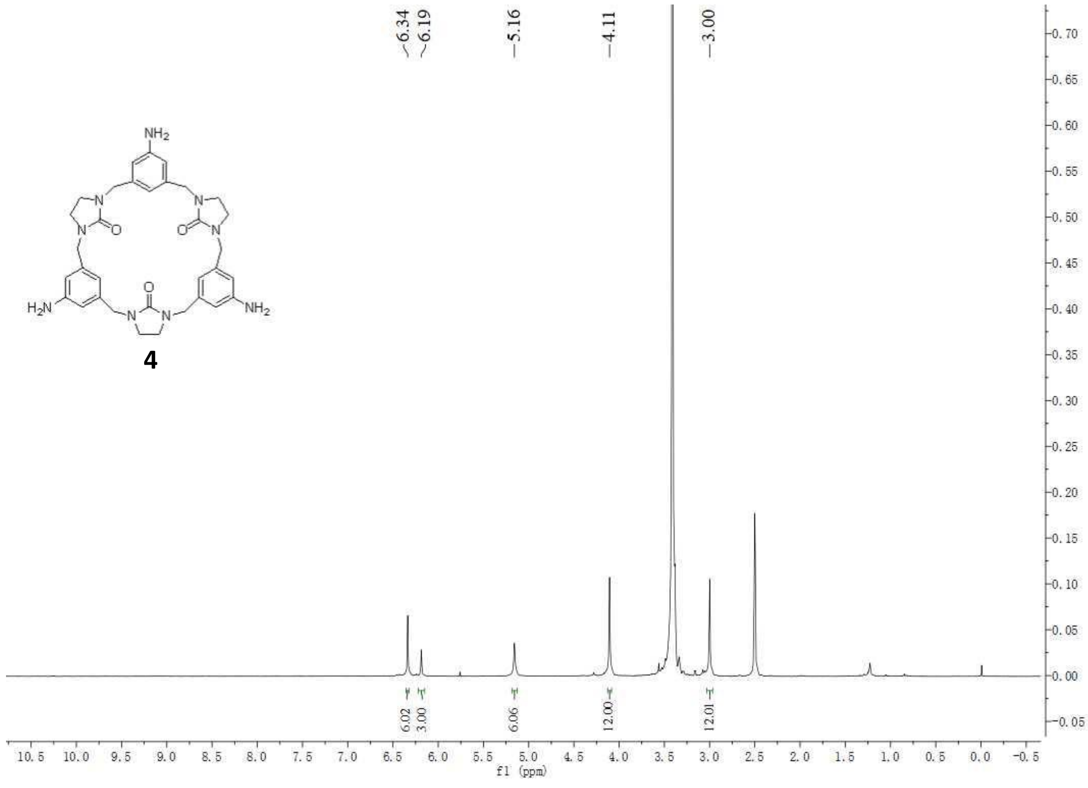

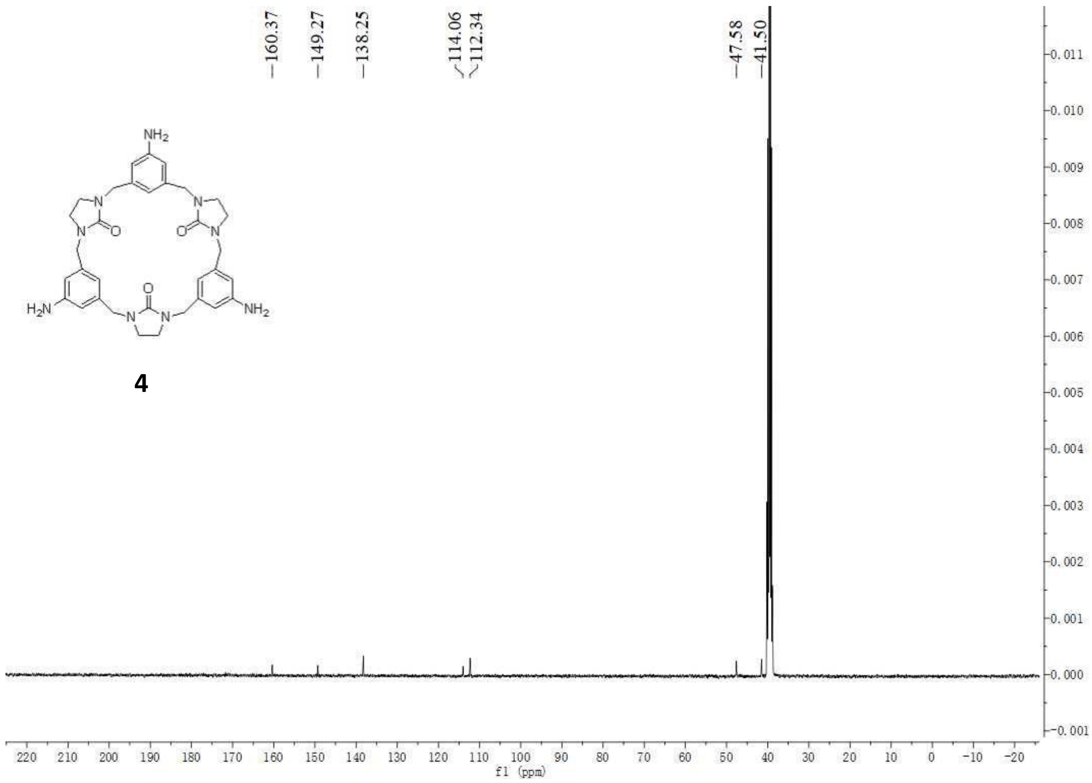

Figure S7: ^{1}H NMR spectrum (400 MHz, DMSO-d_6) and ^{13}C NMR spectrum (100 MHz, DMSO-d_6) of aminobenzene-containing hemicucurbituril **4**.
